# Mrs. Lucy Broad

**Published:** 1914-04

**Authors:** 


Mrs. Lucy Broad, who will be remembered by the older generation of Buffalo physicians as the proprietor of a drug store at 17 South Division street, and who was popularly known as Dr. Broad, died in March, as the result of accidental burns, aged 83. She had retired from active life many years ago, and had engaged in many private philanthropies.

